# Detection and Sequencing of Multiple Human Norovirus Genotypes from Imported Frozen Raspberries Linked to Outbreaks in the Province of Quebec, Canada, in 2017

**DOI:** 10.1007/s12560-021-09507-8

**Published:** 2022-01-23

**Authors:** Philippe Raymond, Sylvianne Paul, André Perron, Christian Bellehumeur, Émilie Larocque, Hugues Charest

**Affiliations:** 1 Canadian Food Inspection Agency (CFIA), Saint-Hyacinthe Laboratory - Food Virology, Saint-Hyacinthe, QC Canada; 2 Laboratoire de santé publique du Québec et Université de Montréal, département de microbiologie, infectiologie et immunologie, Montréal, QC Canada

**Keywords:** Norovirus, Raspberries, RNA extraction, Silica, Confirmation, NGS

## Abstract

Human noroviruses are among the main causes of acute gastroenteritis worldwide. Frozen raspberries have been linked to several norovirus food-related outbreaks. However, the extraction of norovirus RNA from frozen raspberries remains challenging. Recovery yields are low and PCR inhibitors limit the sensitivity of the detection methodologies. In 2017, 724 people from various regions of the Province of Quebec, Canada, were infected by noroviruses and the outbreak investigation pointed to frozen raspberries as a putative source. A new magnetic silica bead approach was used for the extraction of viruses from different outbreak samples. The RNA extracts were tested by reverse transcription-quantitative polymerase chain reaction (RT-qPCR) and five samples were confirmed positive for norovirus by RT-qPCR amplicon sequencing. A multiplex long-range two-step RT-PCR approach was developed to amplify norovirus ORF2 and ORF3 capsid genes from the positive frozen raspberry RNA extracts and other sequencing strategies were also explored. These capsid genes were sequenced by Next-Generation Sequencing. Phylogenetic analyses confirmed the presence of multiple genotypes (GI.3, GI.6, and GII.17) and intra-genotype variants in some of the frozen raspberry samples. Variants of genotype GI.3 and GI.6 had 100% homology with sequences from patient samples. Similar strains were also reported in previous outbreaks. Confirmation approaches based on sequencing the norovirus capsid genes using Next-Generation Sequencing can be applied at trace level contaminations and could be useful to assess risk and assist in source tracking.

**Supplementary Information:**

The online version contains supplementary material available at 10.1007/s12560-021-09507-8.

## Introduction

Human norovirus (HuNoV) is one of the leading causes of acute gastroenteritis. HuNoV is transmitted mainly via the fecal–oral route. In fact, HuNoV can persist in an infectious state for prolonged periods of time in the environment, in water and in food [reviewed in Cook et al. (2016)]. In developed countries, contaminated vegetables, fruits, cereals, sprouts, herbs and spices have been associated to most HuNoV outbreaks (Boqvist et al., 2018; Bozkurt et al., 2020). HuNoV contaminated berries were involved in 46 foodborne outbreaks with over 15,000 cases reported globally between 1983 and 2018 (Bozkurt et al., 2020). Interestingly, frozen raspberries were involved as a food vehicle for more than 80% of documented HuNoV outbreaks (Boqvist et al., 2018; Bozkurt et al., 2020). More recently, several HuNoV outbreaks associated to frozen raspberries were also reported in Canada and in the US (CDC, 2018; Fiset et al., 2018).

**Fig. 1:**
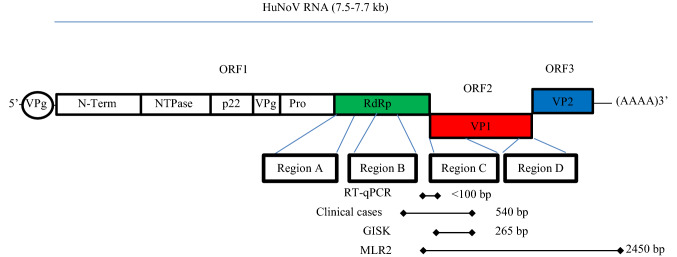
Schematic localization of the norovirus regions targeted by the different sequencing strategies. Localization of the three ORFs and the corresponding major VP1, minor VP2 capsids and RdPd regions. The regions targeted by the RT-qPCR, MLR2 and GISK assays in the current study and in the clinical cases associated to the 2017 outbreak are represented with diamond arrows. HuNov regions A–D are frequently sequenced to determine viral genotypes and subtypes

Noroviruses are small, non-enveloped viruses with a positive polar single-stranded RNA genome of 7.5–7.7 kb which belong to a genetically diverse group of viruses of the *Caliciviridae* family. The virus RNA-dependent RNA polymerase (RdRp) and other non-structural proteins are cleaved from a polyprotein encoded by the ORF1 (Fig. 1). The major structural capsid protein (VP1) and the minor structural capsid protein (VP2) are encoded within the ORF2 and ORF3, respectively (Vinje, 2015). There are 10 distinct norovirus genetic groups (Chhabra et al., 2019). Norovirus genogroups I, II, IV, VIII, and IX infect humans. Genogroups are sub-classified into genotypes based on the VP1 amino acid sequences and in P-types based on the RdPd typing (Chhabra et al., 2019).

There is currently no culture method established to confirm the presence of infectious HuNoV at the levels found in food commodities. Detection of HuNoV relies on viral recovery from food matrix, extraction of RNA and reverse transcription-polymerase chain reaction (RT-PCR) amplification methodologies (Vinje, 2015). The extraction of HuNoV from contaminated frozen raspberry matrices are associated with low recovery (2–9%) (Raymond et al., 2021; Summa & Maunula, 2018). Presence of PCR inhibitors is frequently reported in RNA extracts from soft fruits and can lead to false negative or inconclusive results (De Keuckelaere et al., 2015; Fraisse et al., 2017; Raymond et al., 2021; Summa & Maunula, 2018). When the inhibition is above 75%, it leads to an “invalid result” according to ISO 15216-1:2017 (ISO, 2017). Extraction methods with low recovery yields also increase the probability of false negative results when it comes to detecting trace amounts of virus in food products close to the human infectious dose. On the other hand, false-positive results can be associated to reverse transcription quantitative polymerase chain reaction assays (RT-qPCR) (Lin et al., 2014; Stals et al., 2013a, 2013b).

To verify positive HuNoV laboratory results, some authors have suggested confirmation of RT-qPCR results by repeating the RT-qPCR assay targeting another region of the virus genome, sequencing the RT-qPCR amplicons or sequencing the full genome (De Keuckelaere et al., 2015; Stals et al., 2013b). Sequencing long virus genome regions from contaminated food to support traceback analysis remains challenging. There is currently no harmonized confirmation approach in food virology to support food safety investigations. We hypothesized that the amplification of a specific 2.4-kb fragment complementary DNA spanning the HuNoV minor and major capsid genes from trace amounts of virus prior to next generation sequencing (NGS) would provide sufficient sequence coverage breadth to characterize HuNoV present in a frozen raspberry sample.

A magnetic silica bead (MSB) approach for the extraction of norovirus was recently developed (Raymond et al., 2021). In this study, its application during HuNoV outbreak investigations linked to contaminated frozen raspberries in Canada, in 2017, is presented. A new sequencing approach that allows the amplification and genotyping of HuNoV extracted from food at trace amounts was also developed. HuNoV sequences obtained by NGS from some of the frozen raspberry samples were identical to those found in clinical samples associated to the outbreaks. The performance of these extraction and sequencing methodologies is presented and discussed.

## Methods

### Virus Stock

HuNoV positive emesis and stool specimens were provided by the British Columbia Centre for Disease Control (BCCDC). The preparation of HuNoV from clarified 10% emesis and stool samples was performed as described by Raymond et al. (2021). RNA was extracted from aliquots of viruses using the RNeasy extraction kit (QIAGEN, Canada) and quantified by RT-qPCR as described in section ‘RNA Extraction’ and ‘RT-qPCR’. HuNoV GII.4 strain CFIA-FVR-019 and GI.5 strain CFIA-FVR-022 were used to estimate the limit of detection (LOD).

### Artificial Contamination of Frozen Raspberries

Frozen raspberries from bags labeled as whole individually quick frozen (IQF) collected at local stores were used to prepare 25 g subsamples in order to perform artificial contamination experiments.

Aliquots of clarified 10% stool sample (HuNoV GII.4 CFIA-FVR-019) were prepared as described before (Raymond et al., 2021). They were vortexed 2 s and diluted in PBS to a final volume of 100 µl per subsample at the genomic equivalent copy (gEq) level needed. Frozen raspberries were spiked as described before (Raymond et al., 2021). Non-spiked frozen raspberries were included in each extraction batch as negative controls. The amount of virus in the 100 µl inoculum was assessed in parallel by extracting the total RNA using the RNeasy kit (QIAGEN) followed by a RT-qPCR assay (see ‘RNA Extraction’ and ‘RT-qPCR’ sections).

### Frozen Raspberry Samples from the Outbreaks

Whole and crumbled IQF raspberry samples under voluntary hold, from recall or associated with outbreak investigations were collected at multiple distributors’ sites and in one childcare centre by inspectors from the Ministère de l’Agriculture, des Pêcheries et de l’Alimentation du Québec (MAPAQ) and the CFIA, in the Provinces of Quebec and British Columbia. Each sample corresponded to a different lot number. The samples were collected in subunits of various sizes ranging from 100 g to 10 kg and sent to the laboratory for testing. From each subunit, a 25 g subsample was prepared to extract the viral RNA except in one instance, where four 25 g subsamples were prepared for each subunit (CFIA-FV-340). A total of 134 subsamples were tested during this study.

### RNA Extraction

Viral RNAs were extracted from frozen raspberries using the MSB method as described in Raymond et al. (2021). Briefly, 40 ml of 150 mM Bis–Tris-Propane buffer pH 8 (Sigma-Aldrich) was used to elute HuNoV from the food matrix (25 g). After elution at pH 8, the eluate was clarified by centrifugation (3500×*g* for 10 min) and pectinase was added. Magnetic silica fine beads (AccuNanobeads, Bionneer), ascorbic and malic acid (Sigma-Alrich) were then added to the supernatant. The pH was lowered at pH 3 with HCl to maximize virus attachment to the beads. The concentrated virus was eluted by increasing the pH to 7–9. The total RNA was extracted by using the RNeasy Qiacube kit supplemented with DNase 1 as recommended by the manufacturer (QIAGEN).

### RT-qPCR

Primer and probe sequences used in this study and references can be found in Table 1. RT-qPCR assays were performed using 5 µl of RNA extracts either on the MxPro system (Stratagene, CA, USA) or the Quantstudio 6 system (Thermo Fisher, Canada) as described previously (Raymond et al., 2021). Briefly, the HuNoV GII RT-qPCR was performed using Qnif2 and COG2R primers and the probe Qnifs (Table 1) and the RNA UltraSense™ One-Step Quantitative RT-PCR System (Thermo Fisher) following the procedure and cycling parameters described in ISO/TS 15216-1:2017 (ISO, 2017). The HuNoV GI RT-qPCR was performed using Qnif4 and NV1LCR primers (Da Silva et al., 2007; Svraka et al., 2007) with the TM9 probe (Hoehne & Schreier, 2006) and the TaqMan™ Fast Virus 1-Step Master Mix (Thermo Fisher). Virus gEq quantification was determined using a calibration curve ranging from 5 to 1.57*10^4^ gEq/µl and generated with in vitro RNA transcripts (Table 1) containing target sequences for HuNoV GI, and GII with an insert as previously described (Raymond et al., 2021). Quantitative synthetic norovirus GII RNA ATCC VR-3235SD™ and GI RNA ATCC VR-3234SD™ (Cedarlane, Canada) at 200 gEq/µl were also used as RT-qPCR external control during the 2017 outbreaks. One undiluted and 1/10 diluted replicate per subsample were tested while ATCC controls and each calibration curve concentrations were run in triplicate. Three no template RT-qPCR controls (NTC) were also included in each run.

**Table 1:** Primers, probes and RNA transcripts used in this study

Method	Primer, probe, transcript name	Binding position and orientation^a^	Sequence 5′–3′	References
*Norovirus GI*				
RT-qPCR, qPCR	Qnif4	Fw 5291	CGC TGG ATG CGN TTC CAT	Vilarino et al. (2009)
RT-qPCR, qPCR	NV1LCR	Rev 5376	CCT TAG ACG CCA TCA TCA TTT AC	Svraka et al. (2007)
RT-qPCR, qPCR	FAM-TM9-MGBNFQ	5321	TGG ACA GGA GAT CGC	Hoehne and Schreier (2006)
PCR	GISKF	Fw 5342	CTG CCC GAA TTY GTA AAT GA	Kojima et al. (2002)
PCR	GISKR	Rev 5671	CCA ACC CAR CCA TTR TAC A	Kojima et al. (2002)
*Norovirus GII*				
RT-qPCR, qPCR	Qnif2	Fw 5012	ATG TTC AGR TGG ATG AGR TTC TCW GA	Loisy et al. (2005)
RT-qPCR, qPCR	FAM-QNIFS-BHQ-1	5042	AGC ACG TGG GAG GGC GAT CG	Loisy et al. (2005)
RT-qPCR, qPCR	COG2R	Rev 5100	TCG ACG CCA TCT TCA TTC ACA	Kageyama et al. (2003)
*MLR2*				
MLR2	Qnif2	Fw 5012	ATG TTC AGR TGG ATG AGR TTC TCW GA	Loisy et al. (2005)
MLR2	Qnif4	Fw 5291	CGC TGG ATG CGN TTC CAT	Vilarino et al. (2009)
MLR2	GI5291-17F	Fw 5291	CGC TGG ATG CGN TTC CA	This study
MLR2	R1PCRTRX30	Rev Tx30SxN	GAC TAG TTC TAG ATC GCG AGC GG	This study
MLR2	Tx30SxN	3′ tail	GAC TAG TTC TAG ATC GCG AGC GGC CGC CCT TTT TTT TTT TTT TTT TTT TTT TTT T	Katayama et al. (2002)
*RNA transcript standard*^b^				
Rt-qPCR	HuNov GI transcript	5289–5377	GGGCGAATTGGGTACGATCGATGCGGCCTCGATATCCGCTGGATGCGCTTCCATGACCTCGGATTGTGGACAGGAGATCGCGATCTTCTGCCC*ACTGAGGGTTGCGTTAGACGGGCGACAGATCGT*CGAATTCGTAAATGATGATGGCGTCTAAGGA	Raymond et al. (2021)
RT-qPCR	HuNov GII transcript	5012–5101	GGGCGAATTGGGTACGATCGATGCGGCCTCGAATTCATGTTCAGATGGATGAGATTCTCAGATCTGAGCACGTGGGAGGGCGATCGCAATCTGGCTCCCAGT*ACTGAGGGTTG*TTTGTGAATGAAGATGGCGTCGAA	Raymond et al. (2021)

^a^The binding position is based on HuNoV GI Norwalk virus (M87661) and HuNoV GII Lordsdale virus (X86557). The RIPCRTRX30 reverse primer bind to the Tx30SxN 3′ tail. *Fw* forward; *Rev* reverse

^b^The RNA transcript inserts are underlined and italicized

In addition, cDNAs were synthesized from selected positive RNA extracts with the RT Maxima H minus polymerase (Thermo Fisher) using the oligonucleotide (dT)_18_ following the manufacturer’s recommendations. They were amplified with the Platinum Taq (Thermo Fisher) using the GISKF/GISKR primers targeting HuNoV GI region C of the VP1 gene (265 bp, Fig. 1) as described in Kojima et al. (2002).

### Multiplex Long-Range Two-Step RT-PCR (MLR2)

A multiplex long-range two-step RT-PCR protocol was developed for amplification of the HuNoV ORF1 and ORF2 regions prior to sequencing. The Qnif4 and Qnif2 forward primers used in the RT-qPCR detection were also used in the MLR2 protocol (Table 1). The R1PCRTRx30 reverse primer developed in this study anneals to the Tx30SxN reverse transcription primer (Katayama et al., 2002) and was designed using Primer3plus (https://www.bioinformatics.nl/cgi-bin/primer3plus/primer3plus.cgi) (Rozen & Skaletsky, 2000). The R1PCRTRx30 primer was blasted in NCBI (https://blast.ncbi.nlm.nih.gov/Blast.cgi) to verify the absence of homologies with HuNoV. The Qnif4, Qnif2 and R1PCRTRX30 primer combination efficiency was verified using a collection of 20 reference clinical samples from the BCCDC (Supplementary Table 2). A modified Qnif4 forward primer (GI5291-17F) based on the Sanger sequence obtained from patient samples in the outbreaks (see section ‘Sequencing’) was also tested. The GI5291-17F design was based on the Qnif4 primer minus the 3′ T. The sequence was blasted in NCBI to evaluate its specificity. The GI5291-17F primer RT-qPCR efficiency was compared to the Qnif4 primer using three reference samples from the BCCDC (HuNoV GI.4 CFIA-FVR-003, HuNoV GI.5 CFIA-FVR-022, HuNoV GI.6 CFIA-FVR-010). Its efficiency with the MLR2 method was tested with the CFIA-FV-0340 and CFIA-FV-0521 samples and the reference strain CFIA-FVR-003 only.

Complementary DNAs (cDNA) were synthesized in a GeneAmp™ PCR System 9700 thermocycler (Thermo Fisher) using 5 µM Tx30SxN primer, 1 µl of 10 mM dNTP mix, 7 µl RNase free water and 5 µl of the total RNA extract as described before (Parra et al., 2017). The mix was centrifuged briefly, incubated at 65 °C for 5 min and then chilled at 4 °C in the thermocycler. The reaction was mixed with 200 U of Maxima H Minus First Strand cDNA (Thermo Fisher), 4 µl of the reverse enzyme reaction buffer and 40 U of RNasin® Plus Ribonuclease Inhibitor (QIAGEN). The reverse transcription was performed at 50 °C for 30 min and terminated by heating the reaction at 85 °C for 5 min. The entire reverse transcription reaction (20 µl: four reactions of 5 µl) was amplified using a long-range multiplex PCR. Each long-range multiplex PCR was performed in the thermocycler using 5 µl of the reverse transcription reaction and primers Qnif4, Qnif2 and R1PCRTRX30 at 0.5 µM each with the Platinum SuperFi polymerase kit following the manufacturer’s recommendations (Thermo Fisher). After an initial denaturation step at 98 °C for 30 s, the cDNA was amplified with 35 cycles of 10 s at 98 °C, 30 s at 60 °C and 90 s at 72 °C followed by a final elongation at 72 °C for 5 min. Amplicons were cooled down to 4 °C and stored at − 20 °C until needed.

In order to identify the positive amplification products, a real-time PCR (qPCR) was performed on the MxPro system with a 1 µl aliquot of the MLR2 products using the primers and probes described above for HuNoV GI and GII RT-qPCR and the Taq platinum PCR kit following the manufacturer’s recommendations (Thermo Fisher). The amplification was performed with an initial denaturation at 95° for 2 min followed by 35 cycles of 30 s at 95 °C, 30 s at 60 °C and 1 min at 72 °C.

### Sequencing

Different sequencing strategies were compared.

RT-qPCR amplicon sequencing: Presumptive positive RT-qPCR amplicons (< 100 bp, Fig. 1) and amplicon obtained using the GISKF/GISKR primers (265 bp) were cloned using a TA cloning kit (Thermo Fisher). A fragment of approximately 300 bp and 465 bp was amplified from transformed bacterial colonies using M13 universal primers and purified using the MinElute kit (QIAGEN). Sanger sequencing was conducted at the Centre hospitalier universitaire de Québec (University Laval, Canada). Sequences were trimmed and verified using BioEdit (Hall, 1999). A subsample, and its sample, was confirmed positive when its BLAST result was norovirus in the targeted ORF1 and ORF2 junction region, and that this sequence was not homologous to the ATCC or in vitro RNA transcript controls. Consequently, not all amplicons from the outbreaks were sequenced. Additional RT-qPCR amplicon sequencing was not required for subsamples when its sample was already confirmed positive.

Next-generation sequencing (NGS): RNA extracts from positive subsamples were also analyzed by NGS following the MLR2 amplification (~ 2450 bp, Fig. 1). The MLR2 products were purified using the QIAquick PCR purification kit (QIAGEN). Purified amplicons were quantified using the Qubit dsDNA HS Assay kit (Thermo Fisher) and concentrations were adjusted to 0.2 ng/µl DNA. NGS was performed using the Nextera XT Index kit (V1 and V2 set A), the Nextera XT DNA Library Preparation Kit and the 150-cycle MiSeq Reagent kit v3 following the manufacturer’s recommendations. Twenty-four subsamples including negative controls were multiplexed per run. The pool was spiked with 1% PhiX library as a control for Illumina sequencing runs. Paired-end sequencing was run on a MiSeq benchtop sequencer (Illumina, CA, USA).

Genotyping noroviruses from patient samples: Consensus sequences from multiple clinical cases during the 2017 outbreaks in two distinct administrative regions (about 750 km apart) were provided by the Laboratoire de santé publique du Québec (LSPQ), the provincial public health laboratory. They were generated following the amplification of regions B, and C (540 bp, Fig. 1) and sequenced using Sanger’s method.

### NGS Analysis Pipeline

NGS sequence analysis was performed using CLC Genomic Workbench v12 (QIAGEN). After pairing the reads and trimming adapters, a quality trim limit of 0.1 (modified-Mott trimming algorithm) was applied and reads below 15 bp were discarded. De novo analysis was used to identify contigs using penalties of 2 for mismatch, 3 for insertion and 3 for deletion. The length and similarities fraction thresholds were 0.9. All contigs were compared locally using BLASTn (Blast 1.0) to a curated collection of published norovirus sequences downloaded from the National Center for Biotechnology Information (NCBI) site (Supplementary Table 1 ST1). Scoring parameters were match 2, mismatch -3, existence 5 and extension 2. Contigs associated to noroviruses were analyzed using BLASTn, this time, on the NCBI web site targeting all organisms to find the most similar reference sequence based on the *E*-value (< 1e−8), score (highest) and High Scoring Pair (HSP) length (≥ 250 bp). Scoring parameters were match 1, mismatch -2, existence 5 and extension 2. When the full-length ORF2 and ORF3 norovirus sequences were not identified de novo, the trimmed reads were reference-mapped to the most similar reference sequence identified in the NCBI search. A minimum of ten reads and a contig longer than 250 bp or coverage of the targeted norovirus genome greater than 25% were required for the subsample to be confirmed positive by NGS. Carry-over associated with library indexes were verified during the analyses and sequencing was repeated with new index when required. All HuNoV NGS sequences generated de novo in this study were submitted to GenBank (Supplementary Table 2 ST2).

### Phylogenetic Analysis

The CLC Genomics Workbench’s very accurate progressive alignment setting was used for calculating alignment. Maximum likelihood based phylogenetic trees were constructed using a Neighbor Joining starting tree. The Model Testing tool was used in order to identify the best suitable substitution models for creating a tree. The variable substitution rate selected was a discretized gamma distribution with 4 rate-classes. Topology variation was used in all cases. The reliability of the inferred trees was performed with Bootstrap analysis using 1000 replicates. The genotype of the capsid reference sequences submitted to GenBank was verified using the Norovirus Typing Tool Version 2.0 (Kroneman et al., 2011).

### Limit of Detection and Quantification

The same serial dilution of HuNoV GII RNA extracts from spiked IQF frozen raspberries ranging from 70,000 to 95 gEq per 25 g previously described (Raymond et al., 2021) was used in this study to estimate the LOD of the MLR2 method followed by quantitative PCR (MLR2 + qPCR) and the NGS approach (MLR2 + NGS). A new serial dilution of HuNoV GI (HuNoV GI.5 CFIA-FVR-022) spiked in IQF frozen raspberries and extracted using the MSB methodology was also tested to estimate the HuNoV GI LOD. For both genogroups, the proportion of positive observations out of the five replicate extractions for each concentration was used to assess the probability of detection and calculate the LOD_50_ and LOD_95_ with the PODLOD program (v9) (Wilrich & Wilrich, 2009). The Pearson correlation coefficient was used to analyze the degree of association of the proportion of positive observations between the RT-qPCR and the MLR2 detection methods (MedCalc 17.5.5). The limit of quantification (LOQ) was defined using the RNA transcript calibration curve when the CV > 35%.

## Results

### Limit of Detection

**Fig. 2:**
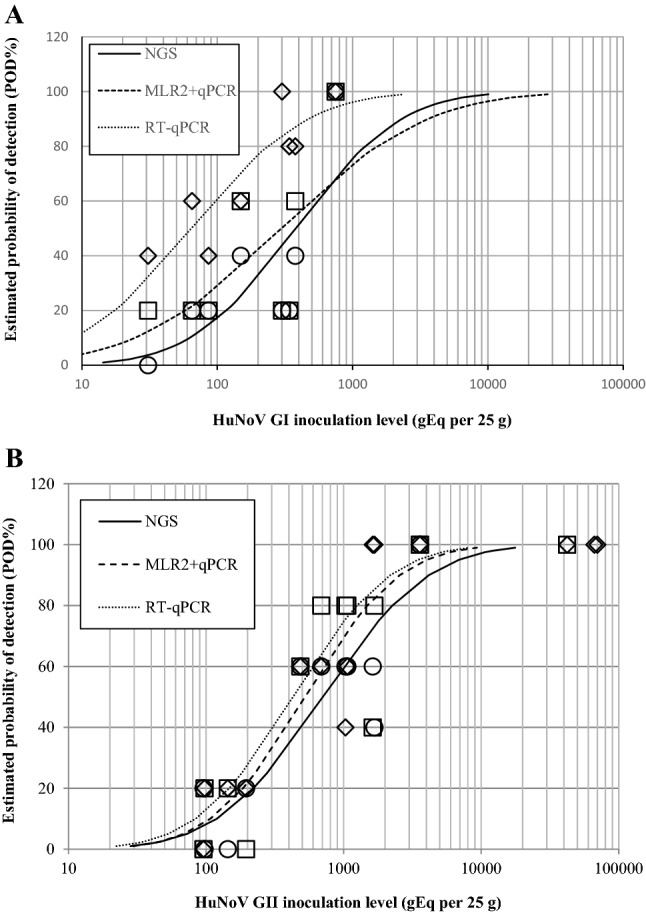
Comparison of the estimated probability of detection (POD) curve of the HuNoV amplification approaches used for detection and sequencing. HuNoV GI.5 (**A**), and GII.4 (**B**) were spiked on frozen raspberry and extracted with the MSB approach. Each observed value represents the ratio of positive result from five extractions tested using the RT-qPCR (◊), MLR2 + qPCR (□) and MLR2 + NGS (○)

The MSB method LOD was evaluated by RT-qPCR as well as by the MLR2 approach followed with either qPCR (MLR2 + qPCR) or NGS (MLR2 + NGS), using RNA extracted from frozen raspberries spiked with a HuNoV GI.5 CFIA-FVR-022 or HuNoV GII.4 CFIA-FVR-019 strains (Fig. 2 and Supplementary Fig. SF1). In the case of HuNoV GI.5, 40 spiked and 8 non-spiked frozen raspberry subsamples were tested. All the 16 samples positive by MLR2 + qPCR were positive by RT-qPCR. Only these 16 positive HuNoV GI.5 subsamples detected by the MLR2 + qPCR analysis were subsequently tested by NGS and 13 were sequenced. The HuNoV GI.5 RT-qPCR LOD_95_ and LOD_50_ were calculated to be at 423 gEq per 25 g (CI95% 262–685) and 98 gEq per 25 g (CI95% 61–159), respectively. The MLR2 + qPCR LOD_95_ and LOD_50_ were slightly higher at 1266 gEq per 25 g (CI95% 749–2140) and 293 gEq per 25 g (CI95% 173–495). The MLR2 + NGS LOD_95_ and LOD_50_ were calculated to be 1663 gEq per 25 g (CI95% 945–2924) and 384 gEq per 25 g (CI95% 218–677).

In the case of HuNoV GII.4, 70 spiked and 13 non-spiked frozen raspberry subsamples were tested. Twenty-five subsamples tested were positive for both RT-qPCR and MLR2 + qPCR. Nine subsamples positive by RT-qPCR were negative by the MLR2 + qPCR while eight subsamples negative by RT-qPCR were positive to the MLR2 + qPCR. Again, only the 33 positive HuNoV GII subsamples detected by the MLR2 + qPCR analysis were sequenced by NGS. Consequently, because of this preselection, there could be a slight bias between the RT-qPCR, the MLR2 + qPCR and the MLR2 + NGS results in the LOD estimates. The MLR2 + qPCR LOD_95_ at 2729 gEq per 25 g (CI95% 1796–4146) was similar to the RT-qPCR LOD_95_ of 2370 gEq per 25 g (CI95% 1542–3642) reported previously (Raymond et al., 2021). Similarly, the MLR2 + qPCR LOD_50_ and the RT-qPCR LOD_50_ were evaluated at 631 gEq per 25 g (CI95% 416–959) and 548 gEq per 25 g (CI95% 357–843), respectively. There was a strong correlation (correlation coefficient = 0.74, *p* = 0.0091) between the ratio of positive HuNoV GII.4 RT-qPCR and the ratio of positive MLR2 + qPCR. Regarding the sequencing results, eighteen HuNoV GII.4 spiked subsamples were confirmed positive by NGS following de novo assembly while 11 could only be confirmed NGS positive by reference mapping. Four subsamples positive by MLR2 + qPCR were not confirmed by MLR2 + NGS. The HuNoV GII LOD_95_ and LOD_50_ for the MLR2 + NGS approach were calculated to be 3612 gEq per 25 g (CI95% 2368–5510) and 836 gEq per 25 g (CI95% 548–1275), respectively. All negative control subsamples were negative by RT-qPCR and MLR2 + qPCR.

### NGS Background

In some of the negative controls that were tested by NGS, a background of reads that mapped to HuNoV GII.4 CFIA-FVR-019 with an average of 106 ± 73 bp (*n* = 10) was measured. This average plus 2 standard deviations was used to establish the mapping thresholds. Carryover contamination was observed in one set of experiments that followed a sequencing run where various strains with over 1 million positive reads each were obtained. It was associated with the shared Nextera index in the negative controls and was detected despite using three standard washes between runs. Following additional system washes, all subsamples from this run were retested with a new index combination and no crosstalk was observed.

### Mismatch and SNP

Fifteen de novo sequences were obtained over 3 NGS runs from the frozen raspberries subsamples spiked with the 10% clarified stool sample CFIA-FVR-019 during the evaluation of the LOD. There was an average of 1.06 ± 0.96 substitutions per 2464 bp, which corresponds to 99.96% homology. Average homologies of 99.97% and 99.94% were observed on the ORF2 (1622 bp) and the ORF3 (807 bp), respectively. By contrast, the sequence CFIA-FVR-019_43523-19_1 obtained from the same 10% clarified stool sample without matrix interference had a total of 1.76 million of norovirus reads, representing 94% of the total read count. Single nucleotide polymorphisms (SNP) were detected in the stool sample with abundance below 8%.

### Norovirus Outbreak

**Fig. 3:**
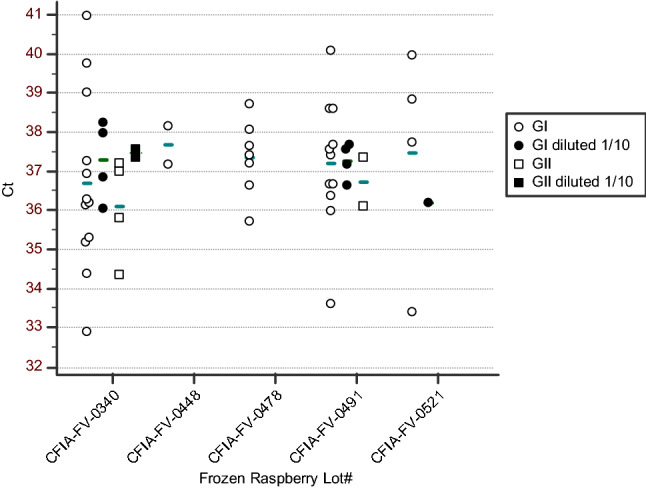
*Ct* values of presumptive positive HuNoV GI and GII samples from contaminated IQF frozen raspberry and raspberry crumble lots. The average *Ct* values for each sample and virus are represented by horizontal lines

In the summer 2017, the CFIA tested eight different samples (lots) of frozen raspberries associated with the various outbreaks occurring in the province of Quebec (Table 2). Six out of these eight frozen raspberry samples were presumptive positive by RT-qPCR. From those samples, there was a total of 39 subsamples presumptive HuNoV positive by RT-qPCR. Four subsamples from two samples were presumptive positive for both norovirus GI and GII by RT-qPCR. Concentrations were below the LOQ (5 gEq per µl) and the calibration curve range for all tested subsamples. The *Ct* values of positive HuNoV GI subsamples were high (32.9–40.8), with an average of 37.3, which would correspond to approximately 2 gEq per RT-qPCR (Fig. 3). The *Ct* values of positive HuNoV GII subsamples were also high (34.4–37.6), with an average of 36.7, representing an equivalent of 0.9 gEq per RT-qPCR based on the standard curve. All calibration curves were within the accepted range of efficiency (90–110%) and precision (*R*^2^ > 0.985). HuNoV was detected mainly in undiluted RNA extracts (36/50) while most diluted RNA extracts were negative. Four subsamples were positive for HuNoV by RT-qPCR when diluted RNA extracts were tested and negative when undiluted RNA extracts were tested. Eight RT-qPCRs were positive with both diluted and undiluted RNA extracts. Based on the *Ct* differences, the RT-qPCR inhibition was greater than 75% in two HuNoV GI subsamples, and was lower than 75% in the other subsamples (5 HuNoV GI and 1 HuNoV GII). A subset of the presumptive positive subsamples was confirmed by Sanger sequencing of the cloned RT-qPCR amplicons. The RT-qPCR amplicon sequences from one presumptive positive (CFIA-FV-0443) were identified as the HuNoV GII ATCC transcript. One subsample was confirmed positive for HuNoV GI and GII while 16 subsamples were confirmed positive for HuNoV GI only. A total of 23 RT-qPCR amplicons were cloned and 141 clones were sequenced. Of these sequences, 23% were aspecific sequences.

**Table 2:** Ratio of positive HuNov frozen raspberry detection and confirmation assay per subsamples from the 2017 outbreaks

Sample ID								
	CFIA-FV-0340^*1^	CFIA-FV-0443	CFIA-FV-0448	CFIA-FV-0478	CFIA-FV-0491	CFIA-FV-0521	CFIA-FV-0571	CFIA-FV-0572
Collection date (yy-dd-mm)	17-06-06	17-06-21	17-06-23	17-07-06	17-07-11	17-07-12	17-07-26	17-07-26
*Detection*								
GI RT-qPCR	12/19	0/30	2/5	7/30	11/30	4/10	0/5	0/5
GII RT-qPCR	4/19	1/30	0/5	0/30	2/30	0/10	0/5	0/5
*Confirmation*								
RT-qPCR Amplicon sequencing	7/7 GI^*2^, 4/4 GII	0/1 GII	2/2 GI	4/4^*2^ GI	2/2 GI^*2^, 2/2 GII	2/2 GI^*2^	NT	NT
Sanger GI region C	1/6	NT	0/2	0/7	NT	2/4	NT	NT
Sanger genotype	GI.6					GI.6		
MLR2 + qPCR	4/7	NT	1/2	1/7	3/12	1/4	NT	NT
MLR2 + NGS GI	2/4	NT	1/1	1/1	2/3	2/2^*3^	NT	NT
MLR2 + NGS GII	1/4	NT	0/1	0/1	0/3	0/2^*3^	NT	NT
NGS genotype	GI.3, GII.17		GI.3	GI.3	GI.3	GI.3,GI.6^*3^		

*NT* not tested

^*1^Five subunits were received from sample CFIA-FV-0340. Four 25 g subsamples per subunit were tested. One extraction failed. Three subunits were positive to HuNoV GI and GII RT-PCR. Two subunits were positive to HuNoV GI RT-PCR only

^*2^Not all subsample RT-qPCR amplicons were verified by sequencing if the sample was already confirmed

^*3^MLR2 repeated twice with a different forward primer

Early in September 2017, the Laboratoire de santé publique du Québec completed sequencing of the partial polymerase and capsid genes (regions B and C Fig. 1) of six clinical samples originating from two of these outbreaks. These samples were identified as genotypes GI.3 (LEOS) and GI.6 (GEOS). The complete GEOS_GI.6-C sequence (540 bp) had 100% homology with NCBI sequences KY653723, MK280882 and MK280882. KY653723 was isolated from a stool sample collected in China in February 2017, while the sequences MK280882 and MK280882 were obtained in 2017 from samples in Australia.

### Region C Sanger Sequencing

**Fig. 4:**
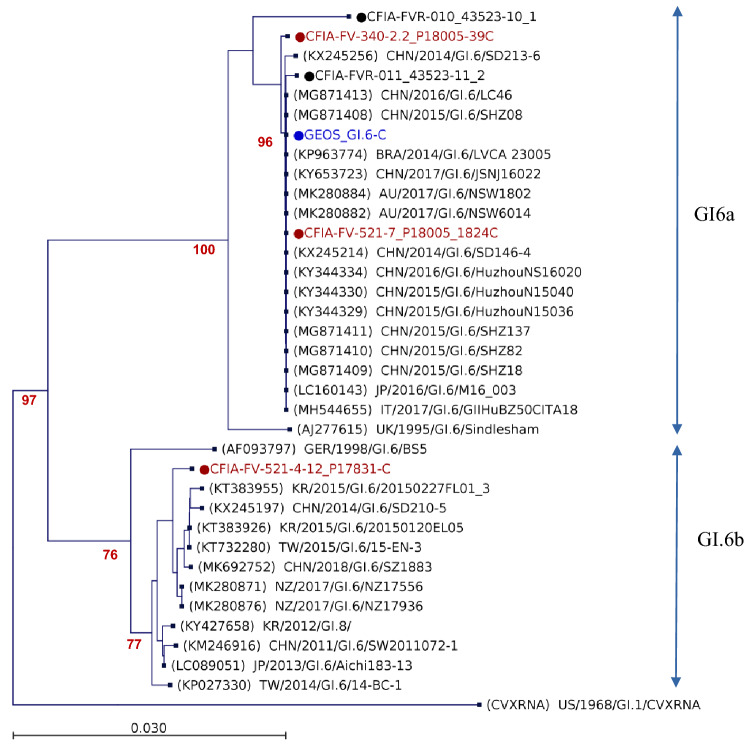
Phylogenetic analysis of HuNov GI.6 ORF1 and ORF2 junction regions (265 bp). The most similar reference sequence and representative sequences, labelled in black, were selected and compared to the HuNoV GI.6 sequence during this study denoted by a dot. Reference sequences were obtained from GenBank and labelled by their accession number, country and year of collection, genotype and ID. HuNov sequence for clinical and frozen raspberry samples collected during this study are labelled in blue and red, respectively. Maximum likelihood phylogenetic three were produced using CLC genomic workbench software with bootstrapping of 1000 replicates, based on the Kimura 80 nucleotide substitution model with variable substitution rate. The bootstrap percentage values are shown for values greater than 70%. The scale bars indicate the number of substitutions per site (Color figure online)

In December 2017, the CFIA laboratory tested 21 HuNoV GI RT-qPCR positive RNA raspberry extracts from the outbreak to enable comparison to clinical sequences using the GISK primer set to amplify the HuNoV region C in the ORF2 (Kojima et al., 2002). Three of these subsample extracts, one from the IQF frozen raspberry sample #CFIA-FV-340 and two from the IQF raspberry crumble sample #CFIA-FV-521, were successfully amplified with the GISK primer and sequenced using Sanger’s method (Table 3). The three sequences were genotyped as HuNoV GI.6. One sequence from the sample #CFIA-FV-340 sequence (CFIA-FV-340-2.2_P18005-39C) and one sequence from the sample #CFIA-FV-521 (CFIA-FV-521-7_P18005_1824C) showed 99.66% and 100% nucleotide homology, respectively, with the partial VP1 GEOS_GI.6-C consensus sequence found in the patient samples over 291 bp (Fig. 4). They belonged to the subcluster GI.6a in the phylogenetic tree. Identical HuNov region C sequence fragments were reported in Australia, Brazil, Italy, Japan and China. Several of these sequences were reported in China’s Shandong province in 2015 (MG871408-MG871413). On the other hand, sequences CFIA-FVR-011_43523-11_2 and CFIA-FVR-010_43523-10_1 obtained from two stool samples collected in Canada in 2010 had also 99.7% and 95.64% homology with the partial RdRp and VP1 GEOS_GI.6-C sequence region over 344 bp, respectively. The other sequence detected in the crumble sample #CFIA-FV-521 (CFIA-FV-521-4-12_P17831-C) belonged to the subcluster GI.6b and showed 88% homology with the GEOS_GI.6-C sequence.

**Table 3:** HuNov sequencing results from the frozen raspberry samples

Outbreak sample ID	RT-qPCR Ct	Sequence #	Sequencing methodology^*1^	Reads after QC and trim (million)^*2^	HuNov sequence length	HuNoV reads	HuNoV sequence coverage depth	Genotype	VP1 MSRS NCBI accession (homology,bp)^*3^	NCBI accession
CFIA-FV-0340	32.9	CFIA-FV-340-1.3_43479-1234	NGS	10.7	2411	2398	71.48	GI.3	LC378990 (99.7%,1685)	MW590338
CFIA-FV-0340	36.98	CFIA-FV-340-1.3_43538-17D	NGS	5.8	2507	2401	68.89	GII.17	KU95534 (99.75%,1623)	MW603758
CFIA-FV-0340	36.2	CFIA-FV-340-2.2_43479-6S	NGS	2.1	1992	275	9.26	GI.3	LC378990 (99,7%,1684)	MW600286
CFIA-FV-0340	36.2	CFIA-FV-340-2.2_P18005-39C	Sanger	NA	311	1	1	GI.6	KX245214 (99.68%,310)	MW603777
CFIA-FV-0448	37.17	CFIA-FV-448-1b_43479-8	NGS	2.7	2410	27,036	795.83	GI.3	LC378990 (99.47%,1685)	MW600279
CFIA-FV-0478	38.06	CFIA-FV-478-13_43479-9	NGS	3.2	2410	5414	160.61	GI.3	LC378990 (99.35%,1684)	MW600284
CFIA-FV-0491	35.97	CFIA-FV-491-16_43538-5	NGS	2.4	2405	3528	105.74	GI.3	LC378990 (99.35%,1686)	MW590347
CFIA-FV-0491	33.59	CFIA-FV-491-23_43538-14	NGS	2.7	2408	12,832	382.85	GI.3	LC378990 (99.35%,1685)	MW590348
CFIA-FV-0521	33.4	CFIA-FV-521-4_43637-6	NGS^*4^	3.4	372	34	6.13	GI.6	KT732280 (98.66%,369)	MW603202
CFIA-FV-0521	33.4	CFIA-FV-521-4_43538-13	NGS	1.1	852	68	5.69	GI.3	LC378990 (99.41%,852)	MW603487
CFIA-FV-0521	33.4	CFIA-FV-521- 4-12_P17831-C	Sanger	NA	334	1	1	GI.6	KY427658 (98.73%,316)	MW603817
CFIA-FV-0521	38.82	CFIA-FV-521-7_P18005_1824C	Sanger	NA	311	1	1	GI.6	KX245256 (99.68%,310)	MW603818

*NA* not applicable

^*1^NGS sequences analysed de novo*,* Sanger sequence from GSK1F/GSK1R cDNA product

^*2^Multiple sequence results obtained from the same extract and the same RT were combined

^*3^The HuNov VP1 most similar reference sequence (MSRS) was identified using Blastn on GenBank

^*4^Modified MLR2 using forward primer GI5291-17F

### NGS Sequencing

Using the MLR2 confirmation approach, the HuNoV detected in the various frozen raspberry RNA extracts collected during the outbreak was further characterized. Thirty-two RNA extracts positive by RT-qPCR were tested using the MLR2 approach and 10 (31%) were found positive by qPCR. From these positive MLR2 amplification products, NGS sequencing datasets were successfully generated for nine subsamples from five different raspberry samples. Three genotypes and two genogroups of HuNoV were identified: GI.3, GI.6 and GII.17. The total read count generated an average of 3.8 million reads, with an average of 5998 reads specific to norovirus and an average sequencing coverage depth of 192×. HuNoV GI.3 and GII.17 sequences were amplified from the contaminated IQF whole raspberries and IQF raspberry crumbles RNA extracts using the MLR2 Qnif4 and Qnif2 forward primers.

The Qnif4 and Qnif2 primers were successful with all BCCDC clinical samples tested (20) including HuNoV GI.6 samples CFIA-FVR-010 (CFIA-FVR-010_43523-10_1) and CFIA-FVR-011(CFIA-FVR-011_43523-11_2) as well as nine other GI and GII samples from stool and emesis specimens (Supplement material ST2). On the other hand, the HuNoV GI.6 sequence from frozen raspberry samples was generated only when a modified Qnif4 forward primer (GI5291-17F) was used. Indeed, a cytosine nucleotide substitution was present at the 3′ end position of the Qnif4 forward primer in the clinical GI.6 sequences and also in two HuNoV GI.6 stool specimens (CFIA-FVR-010 and CFIA-FVR-011) from the laboratory. When analyzed on the NCBI website using the BLASTn algorithm, 33% of the HuNoV GI.6 variants found had the thymine substitution while 67% had a cytosine instead (*n* = 61). No significant similarity was found for the modified Qnif4 primer (GI5291-17F) when the Norovirus taxid:142786 was excluded from the Entrez query. The modified Qnif4 primer (GI5291-17F) was tested and was able to amplify a fragment (CFIA-FV-521-4_43637-6) of the HuNoV GI.6b variant after two attempts from the frozen raspberry crumble sample CFIA-FV-521. The primer did work with sample CFIA-FV-340, but there was no GI.6 sequenced.

**Fig. 5:**
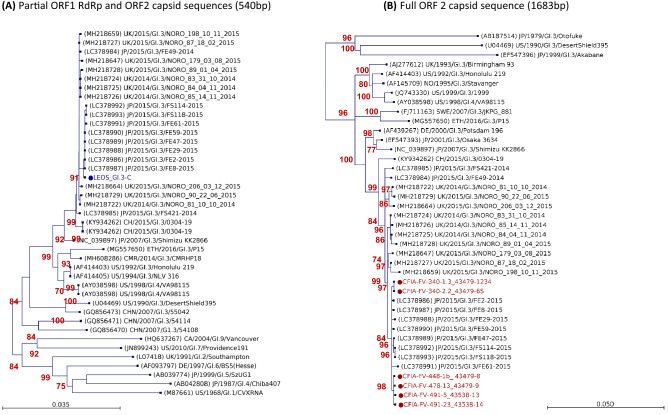
Phylogenetic analysis of HuNoV GI.3 **A** partial ORF1 RdRp and ORF2 capsid regions and **B** full-length ORF2 capsid sequences. The most similar reference sequence and representative sequences, labelled in black, were selected and compared to the GI.3 sequence detected during this study denoted by a dot. Reference sequences were obtained from GenBank and labelled by their accession number, country and year of collection, genotype and ID. HuNov sequences for clinical and frozen raspberry samples collected during this study are labelled in blue and red, respectively. Maximum likelihood phylogenetic tree of the partial ORF1 RdRp and ORF 2 capsid regions (540 bp) and Maximum likelihood phylogenetic tree of HuNoV GI.3 full-length ORF 2 capsid sequences (1683 bp) were produced using CLC genomic workbench software with bootstrapping of 1000 replicates, based on the Kimura 80 nucleotide substitution model with variable substitution rate. The bootstrap percentage values are shown for values greater than 70%. The scale bar indicates the number of substitutions per site (Color figure online)

The 3′ end of the LEOS GI.3 clinical consensus sequences (LEOS_GI.3-C) had 100% homology (344 bp) with sequences from 3 different IQF frozen raspberry samples: CFIA-FV-448, -478 and -491. It had only two synonymous differences with the IQF whole frozen raspberries sample #CFIA-FV-0340 GI.3 sequences over the same region. We further compared the HuNoV ORF2 (1634 bp) and ORF3 (648 bp) capsid nucleotide sequences extracted from the frozen raspberry samples to sequences available on the NCBI database. The GI.3 ORF2 sequences from sample #CFIA-FV-0340 had higher homology (99.45–99.69%) to sequences from a 2015 outbreak in Fukuoka, Japan (LC378986, LC378987, LC378989 and LC 378990) than to the intra-genotype variant found in other IQF frozen raspberry samples (99.08–99.27%) (Fig. 5). Several of the sequence variations observed between IQF frozen raspberry samples CFIA-FV-448, -478, -491 and CFIA-FV-0340 were shared with these Fukuoka sequences. The IQF raspberry crumble sample #CFIA-FV-521 was also positive for HuNoV GI.3, but only 853 bp of the ORF2 was sequenced. The GI.3 ORF2 of sample #CFIA-FV-521 had 99.77% and 99.41% homology to the sample #CFIA-FV-0340 and to the Fukuoka strain LC378990, respectively. Few sequences covering either HuNoV GI.3 ORF2 and ORF 3 or the HuNoV GI.3 ORF3 only were available on the Genbank database. ORF3 sequences from an outbreak in UK in 2014 were the closest homologues, with 99.23% homology to the CFIA-FV-340-1.3_43479-1234 sequence (Supplementary Figs. SF2 and SF3).

**Fig. 6:**
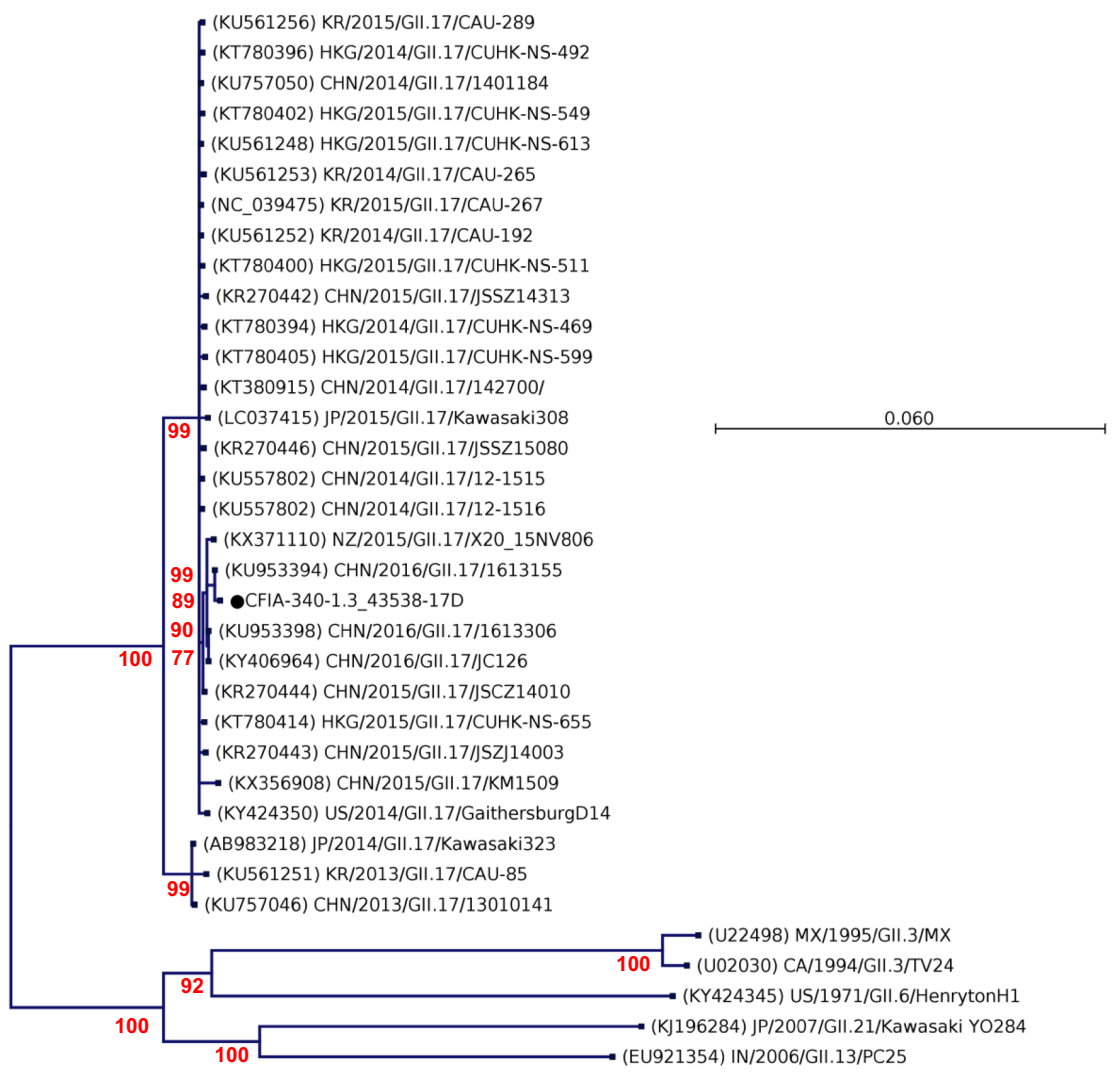
Phylogenetic analysis of HuNoV GII.17 ORF2 capsid sequences. The most similar reference sequence and representative ORF2 sequences, labelled in black, were selected and compared to the GII.17 sequence detected IQF frozen raspberries denoted by a dot. Reference sequences were obtained from GenBank and labelled by their accession number, country and year of collection, genotype and ID. Maximum likelihood phylogenetic three were produced using CLC genomic workbench software with bootstrapping of 1000 replicates, based on the Hasegawa–Kishino–Yano nucleotide substitution model with variable substitution rate. The bootstrap percentage values are shown for values greater than 70%. The scale bars indicate the number of substitutions per site

In addition to HuNoV genotypes GI.3 and GI.6, the frozen raspberry sample #CFIA-FV-0340 was also positive for HuNoV GII.17. To our knowledge, no clinical cases were associated to the HuNoV GII.17 during these outbreaks, although samples from only two outbreaks were characterized by the LSPQ. The #CFIA-FV-0340 GII.17 major capsid protein VP1 nucleotide sequence had three synonymous and one non-synonymous substitutions, the equivalent to 99.75% homology with the GII.17 sequence KU953394 collected in 2016 in Shanghai, China (Fig. 6). This VP1 sequence was associated to a group of 2016 sequences that appears to be in a separate cluster than the earliest, global and emerging sublineages reported by Chan et al. (2017) in their survey of the GII.17 strains circulating between 2014 and 2016 in China (Supplementary material SF4). The first 344 bp of the partial RdRp and VP1 sequence regions had 99.7–100% homology with variants reported in multiple countries, including in the Shandong region, China in 2016 (Supplementary material SF5).

## Discussion

### Norovirus Outbreaks

Between March and August 2017, several HuNoV outbreaks were reported in different regions of Quebec (Table 4) (Fiset et al., 2018). The total of 724 cases reported underestimated the extent of the outbreaks since it did not take into account all cases associated to the suspected source. In addition, it was not possible to assess the extent of secondary transmission in the total number of cases reported (Fiset et al., 2018). Between June and August 2017, the CFIA tested lots of frozen raspberries associated with the various outbreaks such as IQF whole raspberries and IQF raspberry crumble. Two raspberry sorbet samples were also collected during the 2017 outbreaks but were not extracted with the MSB protocol since this extraction method does not perform well with broken down or inappropriately thawed raspberries (i.e. with altered structural integrity) and results were excluded from this study (Raymond et al., 2021). As a result of the outbreak investigation carried out after the confirmation of the presence of norovirus in raspberries in June 2017, the Ministère de l’Agriculture, des Pêcheries et de l’Alimentation (MAPAQ) and the Canadian Food Inspection Agency (CFIA) proceeded, in collaboration with food establishments, to food recalls targeting three Quebec importers sourcing from the same Chinese supplier. At the end of July, the federal government also issued a lookout for the Chinese supplier (Fiset et al., 2018).

**Table 4:** Norovirus outbreaks in the Province of Quebec linked to consumption or handling of frozen raspberries, March–August 2017 [Adapted from ( Fiset et al., 2018)]

Region	Outbreak month	Sick individuals			Location
		Users/Clients	Employees	Total	
Mauricie et le Centre-du-Québec	March–April	204	141	345	Six retirement homes supplied by a common central kitchen
Laurentides	June	187	0	187	Hotel (meeting)
Chaudière-Appalaches	June	26	4	30	Childcare center
Capitale-Nationale	July	7	0	7	Childcare center
	July	46	0	46	Retirement home
Abitibi-Témiscamingue	August	109	0	109	Retirement home
Province of Quebec	Total	579	145	724	

### Viral RNA Extraction and Detection

Frozen raspberries have been associated to several norovirus outbreaks but it remains a challenging food matrix for virus detection due to the low recovery of virus from the food matrices and the presence of RT-qPCR inhibitors (Fraisse et al., 2017; Raymond et al., 2021; Stals et al., 2011b; Summa & Maunula, 2018). In the present study, HuNoV were most often detected in undiluted RNA extracts from frozen raspberry samples when RNA was extracted using the MSB method, an approach that extracts and concentrates norovirus from raspberries based on the variations of the virus surface charges at different pH levels. In contrast, a European survey reported that HuNoV were only detected in spiked frozen raspberry samples when using tenfold diluted RNA extracted with the ISO/TS 15216-1:2013 method (Loutreul et al., 2014). The level of RT-qPCR inhibition associated to both HuNoV GI and GII extracted from frozen raspberries with MSB and detected with the TaqMan Fast Virus 1-Step and the RNA UltraSense kit was previously determined to be 1% (95% CI − 7 to 9) and 58% (95% CI 32 to 83), respectively (Raymond et al., 2021). However, recovery and inhibition could vary substantially between different lots of frozen raspberries, especially if they were thawed multiple times. The RT-qPCR kit had a major impact on the level of inhibition. In this study, the level of PCR inhibition observed with HuNoV GI was limited, as suggested by the RT-qPCR results since most positive results were observed with undiluted RNA extracts. There was no major increase in positive results associated to the diluted extracts excepted for a few samples. However, the presence of the virus at trace amounts could limit the detection of the 1/10 diluted samples and the evaluation of the relative inhibition. We cannot exclude that the HuNoV GII detection was impacted by the RT-qPCR inhibition.

Increasing the number of subsamples extracted increase the probability of detection. However, processing time, cost and resources also increase accordingly. The 50% human infectious dose (HID50) of the HuNoV in susceptible healthy adults varies with the serogroup. The HID50 of the HuNoV Norwalk strain was estimated at 1320 (95% CI 440–3760) genomic equivalent (gEq) in serogroups O and A (Atmar et al., 2014). We reported previously that the MSB HuNoV GI and GII recovery yields range from 2.6 to 5.7% (Raymond et al., 2021). Based on the Poisson distribution (Fig. 2), at the HID50 level, MSB HuNoV GII extractions have to be performed in triplicate in order to generate, with a probability of 95%, a sequence using the MLR2 amplification method based on the current recovery yield. The high number of samples from the outbreaks confirmed positive by NGS can be explained in part by the methodologies used but would not have been achieved without the high number of subsamples tested for each sample. In this study, both the HuNoV GI.5 and GII.4 NGS LOD were evaluated and were below 1000 gEq per 25 g. However, additional experiments to estimate the sensitivity with other genotypes of the NGS approach following the MLR2 amplification are required to confirm this model.

### Confirmation

The probability of non-specific amplification increases with the cycle number during RT-qPCR and accordingly false positives occur more frequently at high *Ct* (~ 40) (Ruiz-Villalba et al., 2017). In this study, the *Ct* values of positive HuNoV GI samples associated to the outbreaks were high and the HuNoV concentrations were below the RT-qPCR LOQ. In order to avoid false positives, it is essential to confirm the RT-qPCR presumptive positive results. Some research groups called only the samples which yield replicate RT-PCR signals as norovirus-positive (Cook et al., 2019). At trace levels, this approach could be prone to false-negative results due to the Poisson distribution. Other groups were able to confirm only a small fraction of RT-qPCR presumptive positive samples by amplicon sequencing (Baert et al., 2011; De Keuckelaere et al., 2015; Stals et al., 2011a). The confirmation process is even more important when using external amplification controls derived from wild type HuNoV sequences. In 2017, the CFIA laboratory was using transcripts carrying inserts for quantification that could therefore be differentiated from wild-type HuNoV to avoid false positive results. However the external controls from ATCC tested in parallel by RT-qPCR contain the ORF-1 and ORF-2 junction sequences of either the HuNov GI.1 or GII.4 (Cedarlane communication), and thus cross-contamination could generate false positive RT-qPCR results. During this outbreak, the RT-qPCR amplicons were cloned and sequenced to confirm the detection results. With this approach, five of six presumptive positive raspberry samples were confirmed by RT-qPCR amplicon sequencing of the junction region and found to be different from the controls. However, this confirmation approach did not provide sufficient information to link the tested samples with the patient samples from the outbreaks because of the amplicon size and the targeted region. The RT-qPCR target area in the junction region is one of the most conserved of the norovirus genome.

### Alternative Sequencing Strategies

Following the 2017 outbreaks linked to raspberries, we evaluated the usefulness of a larger fragment of the genome for sequencing and confirmation. At first, we targeted the HuNoV GI region C on the ORF2 using the GISK primers since it was the initial sequencing region targeted to genotype the clinical cases. HuNoV from only 3 out of 21 raspberry subsamples tested could be typed and were identified as GI.6, one of the two genotypes identified in the patient samples. Indeed, the HuNoV sequence from the raspberry crumble subsamples shared 100% homology with the clinical samples (GEOS). Thus, combined with the outbreak investigation tracing back to the supplier, this raspberry crumble sample had a high probability to be linked to the GI.6 clinical cases. Moreover, these clinical case sequences were also 100% homologous to 2017 clinical case sequences from multiple countries which could suggest a worldwide distribution of this HuNoV GI.6 variant. The small number of HuNoV sequenced from the positive subsamples using this approach could be explained by the low contamination level and sampling error arising from Poisson distributed data. The competition between norovirus strains might also have reduced the capacity of this method to detect the other genotypes identified in the patient samples and in the raspberry samples using the MLR2 NGS approach. The presence of a mismatch on the HuNoV GI.3 sequences at the GISK primer site could also have reduced the amplification efficiency.

Because of the limited success observed with the GISK primer set, we did not attempt to sequence the HuNoV GII region C of the positive outbreak subsamples by Sanger sequencing. Owing to the multiple virus detection assays, we only had a limited amount of residual RNA extract left (< 30 µl) from the positive raspberry subsamples. Like most RNA viruses, norovirus sequences are very heterogeneous. Some authors have suggested using multiple primer sets or performing genome walking to characterize the HuNoV genome (Hasing et al., 2014; Kundu et al., 2013). These strategies were not suitable to the present study because of the trace amount of HuNoV and the limited RNA extract volumes. Moreover, the multiple specific primers required for amplification need frequent updating due to the rapidly evolving nature of HuNoV. Other strategies, like whole transcriptome shotgun sequencing RNA-SEQ or primer-independent NGS, are more suitable for clinical samples rather than food samples. Indeed, the virus concentration in clinical samples ranges from 10^8^ to 10^9^ gEq per g of stool (Fonager et al., 2017). One group reported that RNA samples from HuNov GII.4 inoculated at 1100 copies on celery was extracted and, using an amplification-independent approach, it was sufficient to generate around 8000 HuNoV reads (Yang et al., 2017). On the other hand, Bartsch et al. (2018) also used a metagenomic sequencing approach in an outbreak associated to frozen strawberries contaminated at a level close to 185 gEq per 25 g and were only able to generate 2 reads (151 and 146 bp) of HuNov GII.4 that were successfully associated to a clinical case.

### NGS

We did not attempt to sequence the full virus genome. Even with clinical specimens, the number of specific HuNoV reads could be low (Fonager et al., 2017). Recent genotyping works relied on the viral capsid genes and the capsid hypervariable P2 region to resolve outbreaks with high accuracy (Fonager et al., 2017). Viral capsids are involved in host-receptor interactions and immune responses (Vinje, 2015). Instead, the full virus genomic amplification method was adapted in this study to a semi-specific approach that is more in line with a confirmation assay. The reverse transcription primer, Tx30xN, that combines a poly A tail and an adaptor sequence, has proved to be useful for full viral RNA virus amplification (Katayama et al., 2002; Parra et al., 2017). To remove the potential discrepancy in terms of target sequences between detection and confirmation, both the RT-qPCR and the PCR step of the MLR2 method shared the same forward primers while the MLR2 reverse primer was based on the Tx30xN adaptor. This approach reduced the impact of the sequence variability between the detection and the confirmation primer sets on the sensitivity of the MLR2 amplification. Indeed, we confirmed by qPCR that the MLR2 method had a LOD close to the RT-qPCR when spiked with HuNoV GII. On the other hand, the GI.6 results obtained suggest that this approach could be further improved.

Amplification procedures similar to a long-range two-step RT PCR have been used previously to evaluate norovirus integrity (Li et al., 2014; Wolf et al., 2009). Differences in the sensitivity between RT-qPCR and long-range two-step RT-PCR could be associated to the degradation of longer strands when viral genomes are not protected by capsids. Food could be contaminated with both intact and degraded viruses. Smaller RNA strands are less susceptible to RNase degradation. Fragmented RNA could be positive to RT-qPCR based on small amplicon detection and negative when a longer region is targeted. Accordingly, positive MLR2 amplification provides additional risk information to the RT-qPCR results in terms of virus genome integrity. In addition, as more sequences are reported on GenBank, the full capsid gene sequence will facilitate the investigation of potential contamination sources. Both approaches might be needed during outbreaks. Detection based on a short amplicon could be used to screen multiple subsamples and detect RNA with the highest sensitivity, while a full capsid gene detection and sequencing could be used to confirm the presence of trace levels of intact or partially intact virus genomes and to generate the more in-depth phylogenetic analysis required for source tracking.

The MLR2 amplification process by NGS and de novo sequence assembly and analysis provided enough reads for the majority of the tested subsamples to cover the full HuNoV ORF2 and ORF3 capsid regions. On the other hand, the NGS reads had to be mapped for several spiked subsamples performed at the LOD range. In addition, the average number of HuNoV reads was relatively low compared to the total reads. This is in contrast to the results from feces samples without matrix. The proportion of reads with single nucleotide polymorphism in the de novo sequences decreased as the number of norovirus reads and their coverage increased. During the LOD experiments, an average of 99.97% homology, or 0.5 substitutions per sequence, was observed on the ORF2 sequence between replicates. The substitution rate was sufficiently low to discriminate two different GI.3 strains in the subsamples obtained from the outbreaks. The NGS assay sequencing error rate should be taken into account in the identification of intra-genotype variants. High-throughput sequencing methods are bounded by their technical and theoretical limitations and sequencing errors cannot be completely eliminated. The selection of reverse transcriptase and polymerase enzymes with minimal global substitution error rates is essential. Relative high error rates could be associated to sequencing run carry-over cross contamination when identical adaptor index are used in consecutive sequencing runs (Nelson et al., 2014). Additional stringent washes, replicates as well as using alternate adapter sequences between runs could resolve this issue. Increasing the viral RNA input with higher recovery yields or reducing the background RNA and DNA from the matrix should improve the sequence coverage significantly and the confidence in the quality of the NGS sequences. Limiting the sequencing error rates improves the accuracy of source tracking.

In Denmark, Muller et al. (2015) also found a link between several clinical cases and frozen raspberries. The exact same GI.Pb_GI.6 sequence region of the capsid gene was found in six clinical samples representing five outbreaks that occurred during a period of 6 months with the exception of a single nucleotide polymorphism. According to Parra et al. (2017), comparison of the HuNoV GII.4 NGS sequences with outbreak consensus sequences in the VP1 revealed only a few substitutions, up to 3 nt, among samples resulting from the same outbreak but differed by more than 9 nt between samples collected from different outbreaks. The HuNoV GI.3, GI.6 and GII.17 substitution rate were estimated to be 2.7*10^−3^, 7.0*10^−4^, and 1.7*10^−3^ nucleotide substitutions/site/year, respectively (Parra et al., 2017). These levels correspond to 99.73%, 99.93% and 99.89% homologies in the VP1 sequence for HuNoV GI.3 GI.6 and GII.17, respectively. Samples having variants with similar levels of homology have probably shared a transmission route or vehicle in the recent past.

### Phylogenetic Analysis of the HuNoV Outbreaks

The 2017 outbreaks in Quebec highlight the impact of food pathogens such as HuNoV on public health. The outbreak investigation and traceback analysis were key to the identification of the source of contamination like in previous outbreaks from frozen raspberries reported in Finland (Sarvikivi et al., 2012), Norway (Einoder-Moreno et al., 2016) and in Denmark (Muller et al., 2015). Usually, due to the low level of virus in contaminated food, it is difficult to detect and confirm the presence of viral RNA in the food matrix. In this study, the 100% sequence homology between the samples from patients and the suspected frozen raspberry samples confirmed their association, even more since it involved two genotypes. The phylogenetic analysis carried out with sequences from a longer region of the genome generated by the MLR2 + NGS approach provided additional information on the contamination source. While only GI.3 and GI.6 cases were found in clinical samples, three different genotypes were identified (GI.3, GI.6, and GII.17) in the frozen raspberry samples using this approach. Moreover, as many as four different variants were identified in this study if we include the region C Sanger results. Outbreaks involving multiple genogroups are more frequently associated to contaminated food and water supplies in comparison to transmission from person-to-person (Matthews et al., 2012). Nevertheless, multiple transmission routes or vehicles could be involved.

In a global survey of acute gastroenteritis in children from 2016 to 2020, the GII.4 was the most common reported genotype at 55% (1325 cases), while GI.3 was the most frequently detected GI genotype at 4% (Cannon et al., 2021). Both GI.6 and GII.17 represented 0.98% of the reported genotypes. The new genotype GII.17 known as GII.17 Kawasaki 308-like has emerged in Asia in 2014–2015 and was since detected sporadically outside Asia including Canada (Chan et al., 2017; Hasing et al., 2019). Oysters and other bivalve shellfish appears to be a common vehicles for transmission of the GII.17 viruses (Desdouits et al., 2020; Lu et al., 2016; Rasmussen et al., 2016).

In addition to Canada, Australia and China, sequences with high similarities to those found in the frozen raspberry products in this study were reported in Japan. The similarities of the HuNoV GI.3 intra-genotype variants with the Japanese cases are of interest since the 2017 GI.3 Quebec outbreaks were associated to a single supplier. The 2-year period with the Japanese cases might indicate a longer contamination event or period. The compliance with good agricultural and hygienic practices from farm to fork is essential to prevent outbreaks. In Denmark, following the detection of the same GI.Pb_GI.6 sequences in clinical samples over several months, the legislation was changed to make heat treatment of frozen raspberries compulsory in professional catering establishments (Muller et al., 2015).

In 2017, a co-occurring outbreak involving 15 confirmed cases was reported in Minnesota, USA. After the analysis of raspberry samples, the U.S. Food and Drug Administration identified a location in China. IQF frozen raspberries from Tai’an in the Province of Shandong, China, were added to the Red List of Import Alert 99-35, citing norovirus GII contamination (Entis, 2017). The supplier identified by the FDA during its sampling was not involved in the Quebec outbreaks. On the other hand, since no public sequencing data were available regarding this American case, it was unknown whether the American and the Canadian outbreaks were linked. It is not always possible to generate virus sequences from the trace levels found in food matrices and few organizations are providing food contamination sequence information. The Minnesota cases might share a similar contamination source since the partial VP1 sequences from two HuNoV strains found in frozen raspberries in Quebec in the current study, HuNov GI.6 and GII.17, had 100% homology with sequences reported from the same Chinese province. However, samples sharing high sequence homology do not preclude other outbreak origins. Nevertheless, sequencing the full capsid genes should be promoted as it could be used to facilitate source tracking and prevent additional cases. It adds to the weight of evidence frequently required in order to perform risk analysis and inform risk management actions.

## Conclusion

New methods to extract and sequence HuNoV virus RNA from frozen raspberries were developed and applied successfully with samples associated in 2017 to norovirus gastroenteritis outbreaks in Quebec, Canada. Using this approach, multiple norovirus variants of different genotypes were identified by next-generation sequencing the capsid genes in the samples associated to the outbreaks. Further work is still required to improve the extraction, detection and sequencing of trace amounts of foodborne viruses. As the capacity of regulatory organizations to detect foodborne viruses keeps improving, the ability to link clinical cases to contaminated food products to assess the risk and to perform source tracking will improve as well.

## Supplementary Information

Below is the link to the electronic supplementary material.

Supplementary file1 (DOCX 926 KB)
