# Supplementary material for: Detection and Sequencing of Multiple Human Norovirus Genotypes from Imported Frozen Raspberries Linked to Outbreaks in the Province of Quebec, Canada, in 2017
Source: Food Environ Virol. 2022 Jan 23;14(1):40–58. doi: 10.1007/s12560-021-09507-8 (PMC8881426; doi:10.1007/s12560-021-09507-8)
Supplement: Supplementary file 1 — Supplementary file1 (DOCX 926 KB) [file 12560_2021_9507_MOESM1_ESM.docx]

**Electronic Supplementary Material**

**Supplementary Figure SF1a. Estimated probability of detection (POD) curve and 95 % confidence interval of the HuNoV GII extracted from spiked frozen raspberries using the MSB extraction method and detected by MLR2 followed by qPCR.** Upper (U) and lower (L) POD 95% confidence intervals are represented with dash and dot, respectively. Each observed value represents five extractions.

**Supplementary Figure SF1b. Estimated POD curve and 95 % confidence interval of the HuNoV GII extracted from spiked frozen raspberries using the MSB extraction method and detected by MLR2 followed by NGS.** Upper (U) and lower (L) POD 95% confidence intervals are represented with dash and dot, respectively. Each observed value represents five extractions. NGS assays were performed only on sample positive by qPCR.

**Supplementary Figure SF1c. Estimated probability of detection (POD) curve and 95 % confidence interval of the HuNoV GI extracted from spiked frozen raspberries using the MSB extraction method and detected by RT-qPCR.** Upper (U) and lower (L) POD 95% confidence intervals are represented with dash and dot, respectively. Each observed value represents five extractions.

**Supplementary Figure SF1d. Estimated probability of detection (POD) curve and 95 % confidence interval of the HuNoV GI extracted from spiked frozen raspberries using the MSB extraction method and detected by MLR2 followed by NGS.** Upper (U) and lower (L) POD 95% confidence intervals are represented with dash and dot, respectively. Each observed value represents five extractions. NGS assays were performed only on sample positive by qPCR.


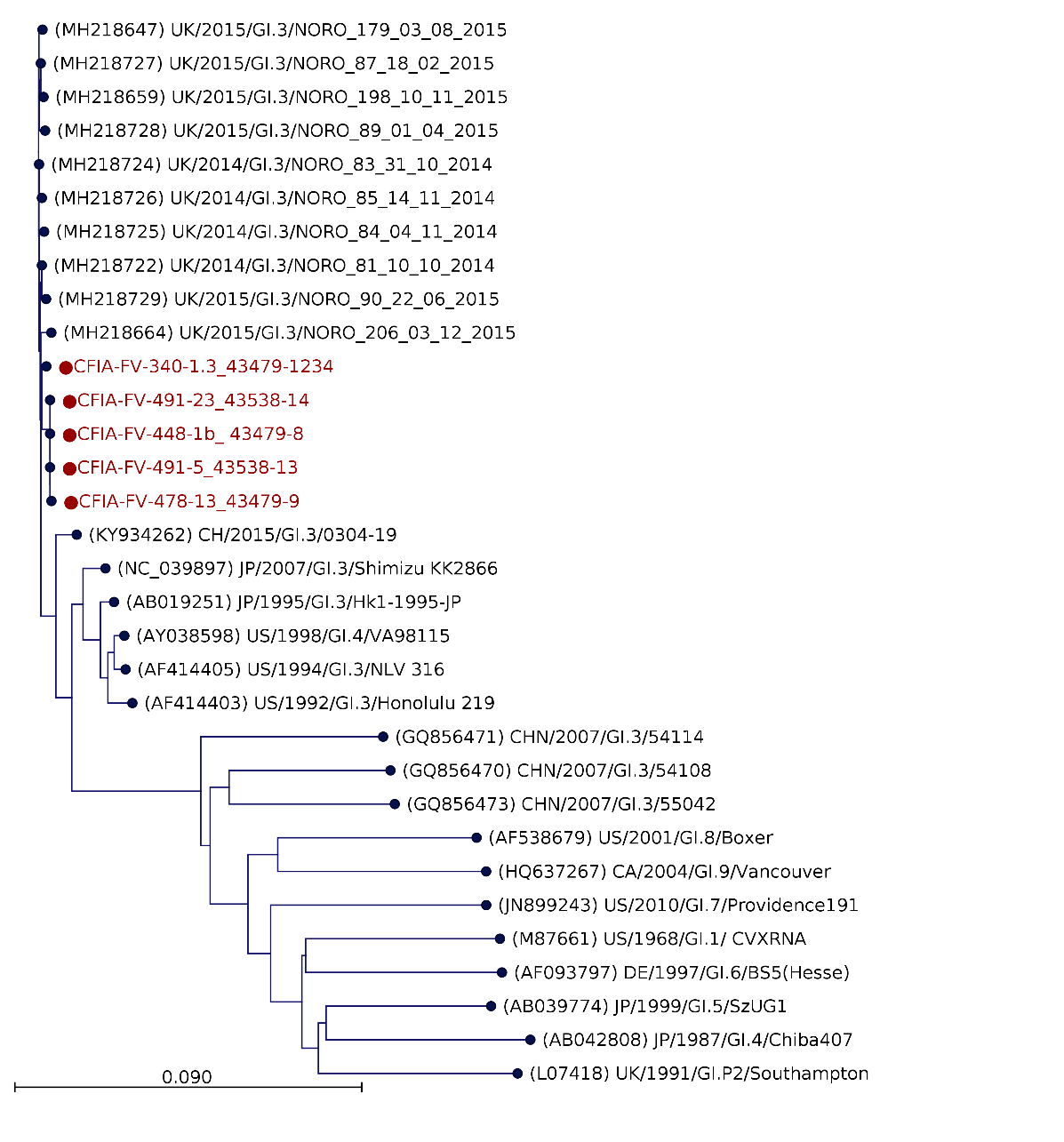


**83**

**99**

**99**

**99**

**98**

**87**

**100**

**98**

**92**

**90**

**75**

**85**

**100**

**94**

**73**

**Supplementary Figure SF2. Phylogenetic analysis of HuNoV G1.3 ORF3 minor capsid sequences.** The most similar reference and representative sequences, labelled in black, were selected and compared to the GI.3 sequence detected during this study denoted by a red dot. Reference sequences were obtained from GenBank and labelled by their accession number, country and year of collection, genotype and ID. Maximum likelihood phylogenetic tree were produced using CLC genomic workbench software with bootstrapping of 1000 replicates, based on the General Time Reversible nucleotide substitution model with variable substitution rate. The bootstrap percentage values are shown for values greater than 70%. The scale bar indicates the number of substitutions per site.


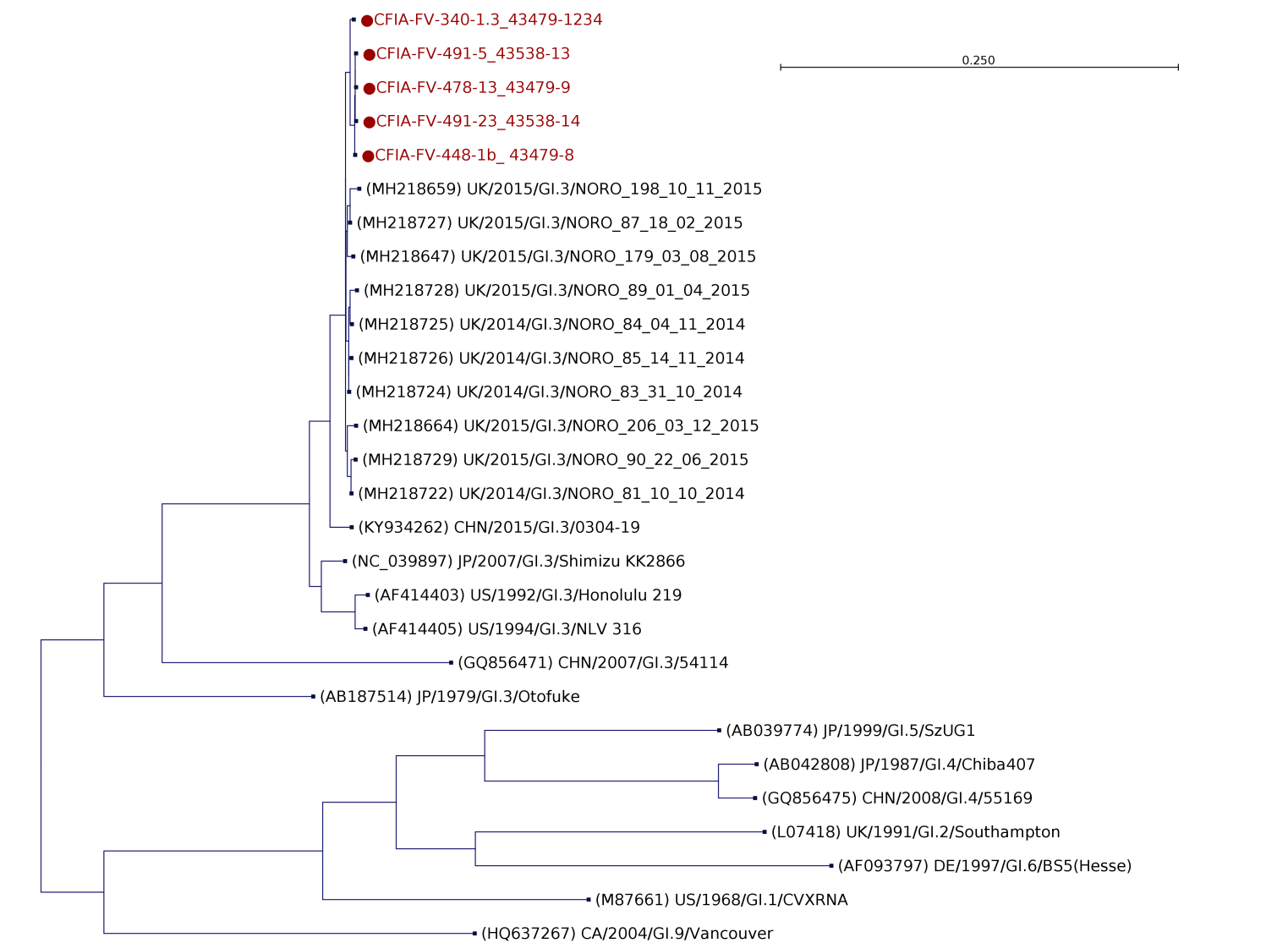


**99**

**80**

**100**

**85**

**99**

**82**

**99**

**93**

**97**

**90**

**75**

**99**

**99**

**100**

**100**

**82**

**100**

**97**

**100**

**87**

**100**

**83**

**100**

**Supplementary Figure SF3. Phylogenetic analysis of HuNoV G1.3 ORF2 and ORF 3, major and minor capsid sequences.** The most similar reference sequence and representative sequences, labelled in black, were selected and compared to the GI.3 sequence detected during this study denoted by a red dot. Reference sequences were obtained from GenBank and labelled by their accession number, country and year of collection, genotype and ID. Maximum likelihood phylogenetic tree were produced using CLC genomic workbench software with bootstrapping of 1000 replicates, based on the General Time Reversible nucleotide substitution model with variable substitution rate. The bootstrap percentage values are shown for values greater than 70%. The scale bar indicates the number of substitutions per site.

**
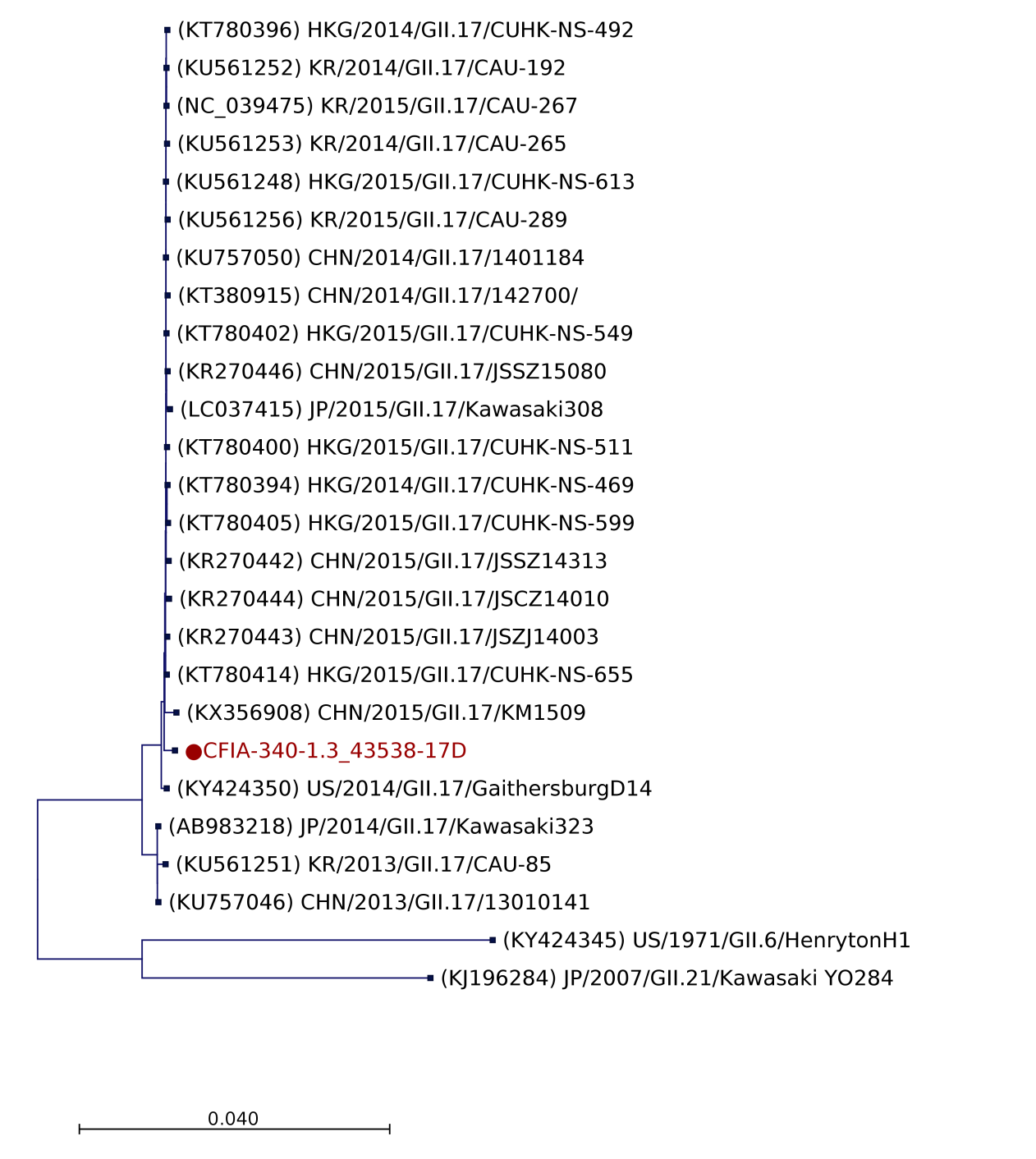
**

**87**

**100**

**97**

**100**

**100**

**Supplementary Figure SF4. Phylogenetic analysis of HuNoV GII.17 ORF2 and ORF3, major and minor capsid sequences.** The most similar reference sequence and representative ORF2 and ORF3 sequences, labelled in black, were selected and compared to the GII.7 sequence detected IQF frozen raspberries denoted by a red dot. Reference sequences were obtained from GenBank and labelled by their accession number, country and year of collection, genotype and ID. Maximum likelihood phylogenetic three were produced using CLC genomic workbench software with bootstrapping of 1000 replicates, based on the General Time Reversible nucleotide substitution model with variable substitution rate. The bootstrap percentage values are shown for values greater than 70%. The scale bars indicate the number of substitutions per site.


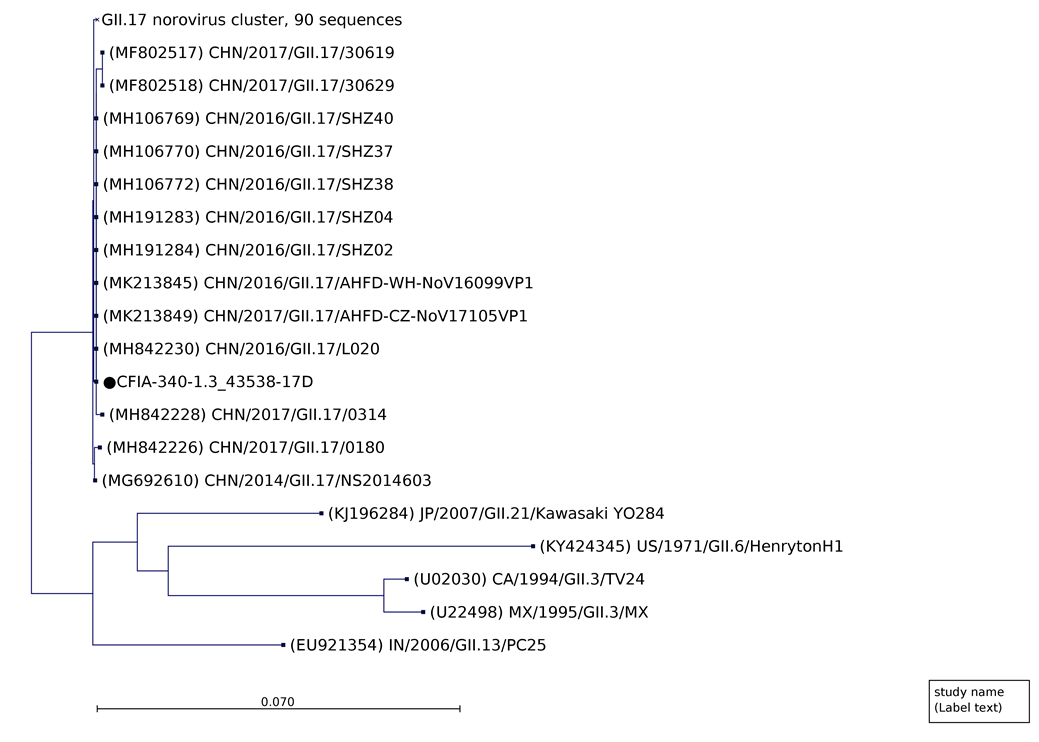


**98**

**88**

**100**

**73**

**98**

**Supplementary Figure SF5. Phylogenetic analysis of HuNoV GII.17 partial ORF1 and ORF 2 junction regions (344bp).** The most similar reference sequence and representative of the ORF1 and ORF2 sequences junction, labelled in black, were selected and compared to the GII.7 sequence detected IQF frozen raspberries denoted by a dot. Reference sequences were obtained from GenBank and labelled by their accession number, country and year of collection, genotype and ID. Maximum likelihood phylogenetic three were produced using CLC genomic workbench software with bootstrapping of 100 replicates, based on the Kimura 80 nucleotide substitution model. The bootstrap percentage values are shown for values greater than 70%. The scale bars indicate the number of substitutions per site.

**Supplementary table ST1. Collection of selected published norovirus sequences found on the National Center for Biotechnology Information (NCBI) site.**

| **Accession** | **Size** | **Description** |
| --- | --- | --- |
| KJ196299 | 7517 | Norovirus GII/Hu/JP/2001/GII.P12_GII.12/Saitama/T18, complete genome. |
| KJ196277 | 7554 | Norovirus GII/Hu/JP/2001/GII.P22_GII.5/Saitama/T49, complete genome. |
| KJ196276 | 7491 | Norovirus GII/Hu/JP/2002/GII.P12_GII.13/Saitama/T80, complete genome. |
| KJ196288 | 7512 | Norovirus GII/Hu/JP/2002/GII.P5_GII.5/Saitama/T52, complete genome. |
| KJ196284 | 7491 | Norovirus GII/Hu/JP/2007/GII.P21_GII.21/Kawasaki/YO284, complete genome. |
| KJ196297 | 7529 | Norovirus GII/Hu/JP/2007/GII.P7_GII.14/Fukuoka/KK282, complete genome. |
| LN854569 | 7579 | Norovirus GII/Hu/NL/2014/GII.21/Groningen, complete genome. |
| NC_029645 | 7313 | Norovirus GIII, complete genome. |
| NC_029647 | 7527 | Norovirus GIV, complete genome. |
| NC_008311 | 7382 | Norovirus GV, complete genome. |
| KF429783 | 7617 | Norovirus Hu/GI.1/8K/1979/USA, complete genome. |
| KF039737 | 7600 | Norovirus Hu/GI.1/CHA6A003_20091104/2009/USA, complete genome. |
| KF306212 | 7740 | Norovirus Hu/GI.2/Jingzhou/2013401/CHN, complete genome. |
| FJ515294 | 7699 | Norovirus Hu/GI.2/Leuven/2003/BEL, complete genome. |
| JQ388274 | 7691 | Norovirus Hu/GI.6/Kingston/ACT160D/2010/AU, complete genome. |
| KU311161 | 7657 | Norovirus Hu/GI.7/AlbertaEI404/2012/CA isolate OU4 non-structural polyprotein gene, partial cds; and major capsid protein and minor capsid protein genes, complete cds. |
| JQ911594 | 7696 | Norovirus Hu/GI/10360/2010/VNM, complete genome. |
| AB187514 | 7746 | Norovirus Hu/GI/Otofuke/1979/JP genomic RNA, complete genome. |
| AB447432 | 7502 | Norovirus Hu/GII-4/Aomori1/2006/JP genomic RNA, complete genome. |
| AB447427 | 7511 | Norovirus Hu/GII-4/Hokkaido1/2006/JP genomic RNA, complete genome. |
| GU017903 | 7529 | Norovirus Hu/GII.14/8533/Maizuru/2008/JPN, complete genome. |
| GU594162 | 7529 | Norovirus Hu/GII.14/8610/Saga/2008/JPN, complete genome. |
| LC145808 | 7482 | Norovirus Hu/GII.2/Ehime5_2014_JP genes for polyprotein, VP1, VP2, complete cds. |
| KJ407074 | 7496 | Norovirus Hu/GII.2/HS255/2011/USA, complete genome. |
| JX846924 | 7527 | Norovirus Hu/GII.3/HK71/1978/CHN, complete genome. |
| GU445325 | 7559 | Norovirus Hu/GII.4/New Orleans1805/2009/USA, complete genome. |
| GQ845367 | 7559 | Norovirus Hu/GII.4/Orange/NSW001P/2008/AU, complete genome. |
| JX459908 | 7564 | Norovirus Hu/GII.4/Sydney/NSW0514/2012/AU, complete genome. |
| JX846927 | 7534 | Norovirus Hu/GII.6/CHDC4073/1984/USA, complete genome. |
| KC576910 | 7441 | Norovirus Hu/GII.6/S9c/1976/SEN, partial genome. |
| KM198561 | 7425 | Norovirus Hu/GII/30303/2009/VNM nonstructural polyprotein and capsid protein VP1 genes, complete cds; and capsid protein VP2 gene, partial cds. |
| KU757046 | 7550 | Norovirus Hu/GII/CN/2013/GII.17/13010141, complete genome. |
| KC662537 | 7492 | Norovirus Hu/GII/Hy-718/KOR, complete genome. |
| LC175468 | 7570 | Norovirus Hu/GII/JP/2016/GII.P16_GII.4_Sydney2012/Kawasaki194 genomic RNA, complete genome. |
| EU424333 | 7420 | Norovirus Hu/GII/Leverkusen267/2005/DE nonstructural polyprotein and capsid protein genes, complete cds; and minor structural protein gene, partial cds. |
| AY772730 | 7579 | Norovirus Hu/NLV/GII/Neustrelitz260/2000/DE from Germany, complete genome. |
| AB220922 | 7533 | Norovirus Hu/Sakai/04-179/2005/JP genes for non structural polyprotein, capsid protein, minor structural protein, partial and complete cds. |
| GU991355 | 7544 | Norovirus Hu/Shanghai/SH312/2009/CHN, complete genome. |
| AB365435 | 7627 | Norovirus Hu/Texas/TCH04-577/2004/US genomic RNA, complete genome. |
| KX907729 | 7648 | Norovirus Hu/USA/2011/GI.P7_GI.7/CS5567, partial genome. |
| KX907730 | 7647 | Norovirus Hu/USA/2014/GI.P7_GI.7/GA5043, partial genome. |
| KX907731 | 7681 | Norovirus Hu/USA/2016/GI.P9_GI.9/SC6350, partial genome. |
| HQ392821 | 7548 | Norovirus pig/GII/Ch6/China/2009, complete genome. |
| JX486101 | 7528 | Norovirus Rn/GV/HKU_CT2/HKG/2011 polyprotein gene, partial cds; and VP1 capsid protein and VP2 minor structural protein genes, complete cds. |
| JX486102 | 7542 | Norovirus Rn/GV/HKU_KT/HKG/2012 polyprotein, VP1 capsid protein, and VP2 minor structural protein genes, complete cds. |
| AY823305 | 7612 | Norovirus swine/GII/OH-QW125/03/US, complete genome. |
| M87661 | 7654 | Norwalk virus nonstructural polyprotein, 58 kd capsid protein, and orf3 genes, complete cds. |
| AF093797 | 7598 | Norwalk virus, complete genome. |
| AB039775 | 7521 | Norwalk-like virus genomic RNA, complete genome, isolate:Saitama U1. |
| AB039778 | 7546 | Norwalk-like virus genomic RNA, complete genome, isolate:Saitama U16. |
| AB039779 | 7546 | Norwalk-like virus genomic RNA, complete genome, isolate:Saitama U17. |
| AB039781 | 7541 | Norwalk-like virus genomic RNA, complete genome, isolate:Saitama U18. |
| AB039782 | 7541 | Norwalk-like virus genomic RNA, complete genome, isolate:Saitama U201. |
| AB039780 | 7524 | Norwalk-like virus genomic RNA, complete genome, isolate:Saitama U25. |
| AB039776 | 7564 | Norwalk-like virus genomic RNA, complete genome, isolate:Saitama U3. |
| AB039777 | 7564 | Norwalk-like virus genomic RNA, complete genome, isolate:Saitama U4. |
| AB039774 | 7700 | Norwalk-like virus genomic RNA, complete genome, isolate:SzUG1. |
| AB045603 | 7521 | Norwalk-like virus genomic RNA, complete genome, specimen_voucher: Gifu'96. |
| AB126320 | 7537 | Norwalk-like virus genomic RNA, complete genome, strain: SW/NV/swine43/JP. |
| AB083780 | 7560 | Norwalk-like virus genomic RNA, complete genome, strain: YURI. |
| AB081723 | 7688 | Norwalk-like virus genomic RNA, complete genome, strain:WUG1. |
| AY126474 | 7311 | Norwalk-like virus isolate Bo/Dumfries/94/UK, complete genome. |
| AY134748 | 7537 | Snow Mountain virus, complete genome. |
| L07418 | 7708 | Southampton virus capsid protein, polyprotein, and ORF genes, complete cds. |
| MW305487 | 7664 | Norovirus GI isolate PNV011143, nonstructural polyprotein (ORF1) gene, partial cds; and VP1 (ORF2) and VP2 (ORF3) genes, complete cds |

**Supplementary Table ST2.** List of strains sequenced by NGS following the MLR2.

| **Sample name** | **Organism** | **Isolate** | **Collected by** | **Collection date** | **Country** | **Isolation source** | **Genotype** | **HuNov**  **sequence length**  **(bp)** | **Genbank**  **Accession**  **numbers** |
| --- | --- | --- | --- | --- | --- | --- | --- | --- | --- |
| CFIA-FVR-001 | norovirus | Human | BCCDC | 2003 | Canada | stool | GI.2 | 2415 | MT745876 |
| CFIA-FVR-002 | norovirus | Human | BCCDC | 2012 | Canada | stool | GI.3 | 2367 | MW558948 |
| CFIA-FVR-003 | norovirus | Human | BCCDC | 2010 | Canada | stool | GI.4 | 2402 | MT750326 |
| CFIA-FVR-004 | norovirus | Human | BCCDC | 2007 | Canada | stool | GI.4 | 2402 | MT874469 |
| CFIA-FVR-005 | norovirus | Human | BCCDC | 2010 | Canada | stool | GI.4 | 2043 | MT756470 |
| CFIA-FVR-006 | norovirus | Human | BCCDC | 2010 | Canada | emesis | GI.4 | 2281 | MT756561 |
| CFIA-FVR-007 | norovirus | Human | BCCDC | 2010 | Canada | emesis | GI.4 | 2408 | MT756627 |
| CFIA-FVR-008 | norovirus | Human | BCCDC | 2007 | Canada | stool | GI.4 | 2402 | MT756629 |
| CFIA-FVR-009 | norovirus | Human | BCCDC | 2010 | Canada | stool | GI.4 | 2037 | MT756631 |
| CFIA-FVR-010 | norovirus | Human | BCCDC | 2010 | Canada | stool | GI.6 | 2401 | MT753150 |
| CFIA-FVR-011 | norovirus | Human | BCCDC | 2010 | Canada | stool | GI.6 | 2400 | MT754179 |
| CFIA-FVR-012 | norovirus | Human | BCCDC | 2007 | Canada | stool | GII.3 | 2511 | MT808057 |
| CFIA-FVR-013 | norovirus | Human | BCCDC | 2004 | Canada | stool | GII.3 | 2510 | MT808058 |
| CFIA-FVR-014 | norovirus | Human | BCCDC | 2008 | Canada | emesis | GII.3 | 2513 | MT808055 |
| CFIA-FVR-015 | norovirus | Human | BCCDC | 2007 | Canada | stool | GII.3 | 2508 | MT808056 |
| CFIA-FVR-016 | norovirus | Human | BCCDC | 2007 | Canada | stool | GII.4 | 2538 | MT754267 |
| CFIA-FVR-017 | norovirus | Human | BCCDC | 2010 | Canada | stool | GII.4 | 2526 | MT754278 |
| CFIA-FVR-019 | norovirus | Human | BCCDC | 2008 | Canada | stool | GII.4 | 2527 | MT754280 |
| CFIA-FVR-020 | norovirus | Human | BCCDC | 2008 | Canada | stool | GII.4 | 2537 | MT754281 |
| CFIA-FVR-021 | norovirus | Human | BCCDC | 2007 | Canada | stool | GII.5 | 2485 | MT754282 |
| CFIA-FVR-022 | norovirus | Human | BCCDC | 2010 | Canada | stool | GI.5 | 2405 | OL345567 |
